# Individual Cell-Level Temperature Monitoring of a Lithium-Ion Battery Pack

**DOI:** 10.3390/s23094306

**Published:** 2023-04-26

**Authors:** Keith M. Alcock, Álvaro González-Vila, Mustehsan Beg, Francisco Vedreño-Santos, Zuansi Cai, Lourdes S. M. Alwis, Keng Goh

**Affiliations:** 1 School of Computing, Engineering and the Built Environment, Merchiston Campus, Edinburgh Napier University, Edinburgh EH10 5DT, UK; 2 Electromagnetism and Telecommunication Department, University of Mons, B-7000 Mons, Belgium

**Keywords:** FOS, FBG, Bragg, lithium-ion, battery, temperature monitoring, guide tube

## Abstract

The work described herein details the deployment of an optical fibre strand with five fibre Bragg grating (FBG) sensors for individual cell-level temperature monitoring of a three-cell lithium-ion battery pack. A polymer guide tube with 3D printed plinths is employed, resulting in high precision temperature readings with an average error of 0.97 °C, 1.33 °C, and 1.27 °C for FBG sensors on each battery cell, surpassing traditional thermocouple and platinum resistance sensors in some circumstances. The temperature response of FBGs positioned between battery cells demonstrates that, in addition to sensing temperature at the cell level, temperature data can be effectively acquired between cells, suggesting that FBGs may be used to monitor the heat radiated from individual cells in a battery pack.

## 1. Introduction

Electrochemical energy storage is rapidly becoming the standard method for electrical energy storage across the world, with various forms of battery storage employed in a wide range of applications. Batteries are classified into two types: primary batteries, which can only be used once and cannot be recharged owing to irreversible electrochemical processes in the batteries, and secondary batteries, or rechargeable batteries, which may be cycled by discharging and recharging. Primary batteries account for a substantial segment of the commercial battery industry; nevertheless, there are drawbacks to using primary batteries, such as the manufacture of vast volumes of non-recyclable materials and the hazardous components in the batteries, which cause environmental issues [1]. The lithium-ion battery (LIB) is a prominent secondary battery due to its multiple advantages, such as high specific energy, high specific power, high conversion rate, and prolonged cycle life. It is extensively used in electric cars and a variety of energy storage systems due to its great electrochemical stability, high energy density, extended battery life, and absence of maintenance needs [2].

The monitoring and regulation of heat generation from an LIB are critical to the battery cell’s longevity and performance. High-temperature exposure and heat production from the cell can cause a variety of degradation processes that result in decreased capacity and power fading. Temperature build-up is created by electrochemical processes within the LIB, and heat generated from entropy change is reversible during operation; however, heat generation caused by charge transfer, ohmic loss, and mass transfer restrictions is irreversible [3], and, therefore, heat build-up occurs.

Individual cell-level temperature monitoring, therefore, is an important concept for the advancement of the LIB pack, particularly the safety aspect. Currently, the LIB temperature is sensed at the module level rather than at the cell level, which is not optimal for the battery thermal management system (BTMS). For commercial vehicles, the primary approach is to measure the temperature at various locales on the surface or tab of LIB cells [4,5]. This temperature in large-format LIBs can differ significantly from the temperature achieved in the LIB core [6], which is the crucial temperature in terms of performance and safety [5]. The overarching battery management system (BMS) protects the LIB cells against abuse, such as over-voltage, under-voltage, over-current while charging or discharging, over-temperature, under-temperature, and cell balancing; however, if abuse conditions occur and a severe temperature gradient is induced under extreme conditions, the separator could shrink or melt, causing an internal short circuit, which can lead to uncontrollable temperature rise, thermal runaway, in the cell [7]. There are many LIB cells in a battery pack. For example, Tesla used more than 7000 18650 LIB cells for the Tesla Model S, and Mitsubishi used 88 large prismatic cells for their Mitsubishi i-MiEV vehicle [7]. With the multitude of LIB cells, it is crucial to obtain sensible temperature data, ideally at the individual cell level.

There are numerous works to obtain battery parameters, such as state of charge, voltage, capacity, and temperature; however, the literature on individual cell-level monitoring of temperature is limited [8,9,10]. A cell-level control technique is presented in [11] by using a control algorithm to bias individual cells differently based on their state of charge, capacity, and internal resistance to mitigate the accelerated ageing effects on weak cells. This research resulted in a longer total pack lifespan. Furthermore, the technique results in a more homogenous distribution of cell capacities at the end of the first life, resulting in more value in the battery pack for second-life applications such as grid energy storage. J. Meyer et al. [12] demonstrate extensive multiplexing of fibre Bragg grating (FBG) sensors in battery systems. To evaluate the strain and temperature from a 13.8 kWh battery pack, 96 FBGs are utilised spanning fourteen fibre optic sensor (FOS) strands. The FBG sensors were calibrated by putting the entire battery pack in a thermal chamber and subjecting it to temperature levels of 15 °C, 30 °C, and 45 °C. For temperature measurement, some FBG sensors are mechanically decoupled by a small tube, while others are directly connected to the cell surface to detect strain. Various abusive circumstances are applied to the cells, including quick charging, overcharging to induce gassing, and cycling at extremely low temperatures to induce lithium plating on the anode. By inserting a nail into a cell, the battery was also vulnerable to short-circuit occurrences that caused thermal runaway [9]. The authors conclude that temperatures of up to 750 °K were recorded during the thermal runaway experiment. The capacity to measure every cell temperature offered a substantially greater maximum cell temperature than that recorded by the BMS, promoting safety, particularly with the ability to measure the temperature across LIB cells [12].

It is predicted that fibre optic sensor approaches will be one of the most prevalent methods for obtaining battery characteristics, owing to the multiplexing capabilities associated with fibre optics, which allow several measurements to be obtained from a single FOS strand [13,14,15]. Many instances of battery surface temperature measurements utilising the FBG sensor can be found in the literature, where the studies typically make use of tubing of some kind to decouple the strain and temperature observations [16,17,18,19,20,21,22]. Thus, it is evident from the literature that the use of FBGs for temperature measurement is an area of great interest where many researchers have successfully utilised the sensing method for the measurement of lithium-ion battery parameters.

The purpose of the works presented herein is to determine the accuracy of the FBG sensors at the individual cell level in comparison to the commercial thermocouple (TC) and Platinum resistance (PT) sensors. An FOS strand with five FBGs is mounted across a three-cell parallel battery pack and discharged at three different conditions: two at constant current (CC), 0.5 C and 2 C, and one at 40-Watts constant power (CW). This is employed to validate the hypothesized ‘guide tube’ method as a simplified mounting strategy for LIB temperature monitoring at the individual cell level. Additionally, two intermediate FBGs situated between the LIB cells are evaluated as a technique for acquiring thermal data from the LIB cells’ radiated heat during discharge.

## 2. Materials and Methods

### 2.1. Fibre Bragg Grating Fabrication

The photoinscription of the FBG array was carried out using a Noria FBG Manufacturing System commercialised by NorthLab Photonics (Nacka, Sweden). The choice of this system was based on its ability to quickly produce several wavelength-shifted FBGs on a single optical fibre, with minimal influence on the fibre strain during manufacturing. This is achieved by combining 193 nm excimer laser inscription for a few-pulse photoinscription (Coherent Excistar XS), the integrated 1D translational stage for precise positioning and the wheel of phase masks for defining each FBG’s central wavelength.

Five FBGs were fabricated in an SMF-28 optical fibre piece. To enhance photosensitivity, the fibre was previously subjected to a hydrogen-loading process. The resulting FBGs were 5 mm long, with phase mask pitches of 1054 nm, 1063 nm, 1070 nm, 1075 nm, and 1082 nm. The laser parameters were set to a pulse energy of 5 mJ and a repetition rate of 50 Hz, and each FBG required a single burst of 100 shots to achieve the desired reflectivity. The optical fibres were finally heated to 100 °C for 36 h to stabilize their working optical response by removing the residual hydrogen content still present within. It is worth mentioning that the refractive index modulation produced on the fibres when using this manufacturing procedure occupies a larger area of the core [23], thus being more uniformly distributed in comparison to FBGs created using other techniques.

### 2.2. Sensor Mounting Method

**Figure 1:**
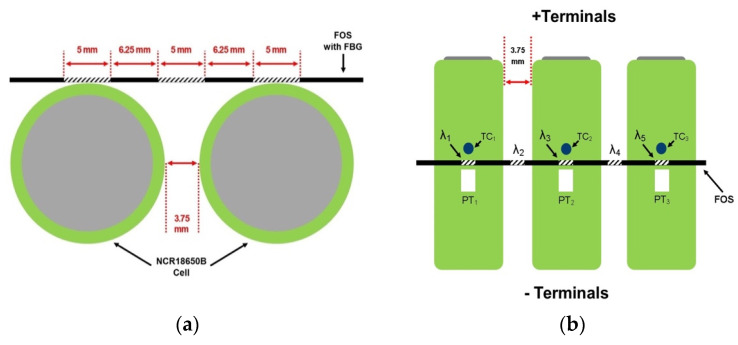
Diagram of the FOS and FBG placement: (**a**) the FOS position across two of the three LIB cells, each cell separated by 3.75 mm, and (**b**) the positioning of the FBGs, 5 mm per FBG with 6.25 mm spacing, one 4-wire PT-100 sensor (PT) and K-type Thermocouple (TC) placed next to each FBG on a cell.

**Figure 2:**
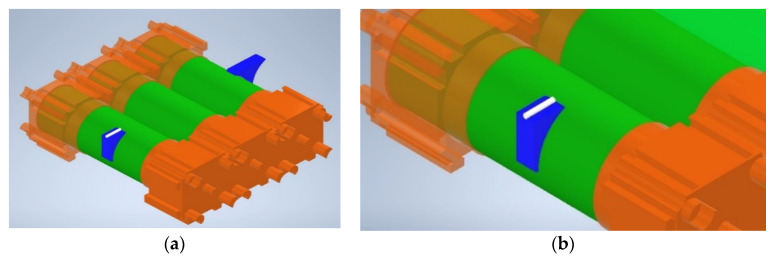
Illustration of the guide tube mounting method. (**a**) The view of the whole assembly where the battery pack is connected using Vruzend battery connectors at both positive and negative terminals, and (**b**) ABS plinth and PTFE guide tube assembly.

The three-cell battery pack was mounted with three separate types of sensors including three K-type TC sensors, three four-wire B-class PT sensors, and five FBG sensors, as shown in Figure 1. Each TC and PT sensor was attached using M Bond 200 adhesive adjacent to the FBG sensor location on each of the three LIB cells. The FBG sensors were positioned as follows: The guide tube technique proposed in [21] was modified to increase cell-surface proximity and reduce longitudinal friction in the FOS by using a micro-polytetrafluoroethylene (PTFE) tube as the guiding structure, allowing the FOS to expand and contract during heating and cooling and, thus, decoupling strain and temperature measurements. As shown in Figure 2, the PTFE guide tube and one end of the FOS are mounted to the LIB cells using 3D-printed acrylonitrile butadiene styrene (ABS) plinths manufactured using a Prusa Mini Plus printer, thus allowing for proximity to the LIB cell surface. To enable the cladding section of the FOS strand to be glued, the plinth on which the FBG 1 was located was longer than the guide tube location. As a result, it offered somewhat additional support.

As theoretically, there is only one contact point because the FBG across each cell is at a tangent to the cylindrical cell surface, a contact length cannot be determined. At defined locations on the FBG, it is feasible to determine the separation distance of the FBG from the LIB surface. Since the FBGs centre point will contact the cell surface, and from this point on, the cell surface will curve away from the FBG, 2.5 mm will be the largest tangential length with the greatest separation from the cell surface. The maximum distance between the FBG and LIB cell surface, as determined by computer-aided design software, is found to be 0.354 mm, which decreases to just 0.087 mm at 1.25 mm from the FBG’s contact point. In addition, the 5 mm FBG covers approximately 5.07 mm in length of the cell circumference. Due to the small distance between FBG and LIB surface, it is assumed that there are no significant losses of heat transfer to the FBG surface, especially since the entire setup is contained in a chamber at a constant ambient temperature.

Additionally, this approach permits two FBGs (FBG 2 and FBG 4) to be placed in the space between the LIB cells, allowing temperature data to be captured from the two neighbouring cells. It is important to note that while this mounting method accounts for the strain induced by temperature on the FOS strand, it does not account for other external influences such as vibration. It is deemed that there is no such outside influence as this study was conducted in a laboratory setting.

### 2.3. Sensor Calibration

The three types of sensors mounted on the cell battery were calibrated before the discharge testing could proceed. The PT and TC sensors were calibrated as follows: The variation in the factory-calibrated thermocouples and platinum resistance sensors at zero degrees Celsius was measured to confirm that they perform within tolerance. They were stored at 0 °C in an ice-filled vacuum flask. After the sensors had attained a steady temperature, the data was logged for 60 min at 1 Hz, and the mean and standard deviation of the sensor responses were determined. The PT sensors indicated a mean of 0.0425 °C with a standard deviation of 0.0183 °C, while the TC sensors showed a mean of 0.0061 °C with a standard deviation of 0.0148 °C. Both sensor types demonstrated good comparability; however, the TC sensors demonstrated to be the closest to zero degrees. It is important to note that the TC sensors have an uncertainty factor of ±0.5 °C and the B-class PT sensors ±0.8 °C.

The FOS, on the other hand, is calibrated in situ, i.e., when it is mounted to the battery pack. A programmed procedure using an ESPEC LU-114 low-temperature chamber (ESPEC Corp., Osaka, Japan) increased the ambient temperature in 5 °C increments from 20 °C to 55 °C (the temperature range of the battery), with each increment continuing until a steady state was attained.

The wavelength shift was measured at 1 Hz for 30 min, and the data was then used to calculate an average wavelength shift for each temperature increment, showing the response of each FBG sensor along the FOS strand. Each FBG sensitivity (k) was determined using the reference wavelengths 1523.157 nm, 1536.11 nm, 1546.062 nm, 1553.229 nm, and 1563.103 nm for FBG 1 to FBG 5, respectively, and the overall sensitivities were then determined using the averaged peak wavelength at each temperature increment. FBG 1 had a sensitivity of 9.463 pm/°C, FBG 2 had a sensitivity of 9.562 pm, FBG 3 had a sensitivity of 9.623 pm/°C, FBG 4 had a sensitivity of 9.688 pm/°C, and FBG 5 had a sensitivity of 9.721 pm/°C. The same technique as in [18] was used to obtain the temperature response of the FBG sensors. Equation (1) shows the temperature value function of the measured peak wavelength, where k is the temperature sensitivity in nanometres per degree Celsius, λ_0_ is the reference wavelength in nanometres, and λ is the measured peak wavelength in nanometres.

T(λ) = (λ − λ_0_)/k (1)

### 2.4. Battery Discharge

Three distinct discharge experiments were carried out, the first two at constant current (CC) and the third at Constant Power (CW). The first test was performed at a discharge rate of 0.5 C, resulting in a CC of ~4.8 A, while the second was performed at a discharge rate of 2 C, resulting in a CC of ~19.2 A. The third was performed at a CW of 40 W, resulting in a current ranging between ~10.55 A and ~16.2 A. The discharged tests are performed from a fully charged battery pack with an open circuit voltage of ~4.18 V to a cut-off voltage of ~2.5 V. The wavelength shifts corresponding to the 5 FBGs across the three-cell LIB pack were obtained using a Micron Optics sm125 FBG interrogator (Luna Innovations, Virginia, USA) and ENLIGHT software (Version 1.18.8). The temperature readings of the thermocouple sensors were recorded using a PicoLog TC-08 data recorder (Pico Technology, Saint Neots, UK) and PicoLog 6 software(Version 6.2.7). The temperature response of the platinum resistance sensors was also captured in the PicoLog 6 software using a PicoLog PT-104 (Pico Technology, Saint Neots, UK) data recorder. The LIB was discharged using a B&K Precision 8610 DC programmable electronic load and charged using a B&K Precision 9202 multirange programmable DC power supply(Yorba Linda, California, USA). Data logging was set to 2 Hz in the ENLIGHT software, and 1 Hz in the PicoLog 6 and B&K Precision software (Version 1.7.0). With all of the data logging equipment placed outside the chamber and the access hole securely sealed, an ESPEC LU114 low-temperature chamber was utilised to maintain a constant ambient temperature of 20 °C ± 1 °C. Additionally, a Faraday cage surrounded the battery pack to shield it from outside influences.

The responses of the FBGs were directly compared to the TC and PT sensors to assess their ability to successfully and precisely record the cell temperatures of the battery pack using the guide tube and plinth mounting technique. Since the three tests have different discharge currents, this induced different levels of heat generation that ultimately caused various temperature changes in the battery pack cells.

## 3. Results and Discussion

### 3.1. Discharge Results

**Figure 3:**
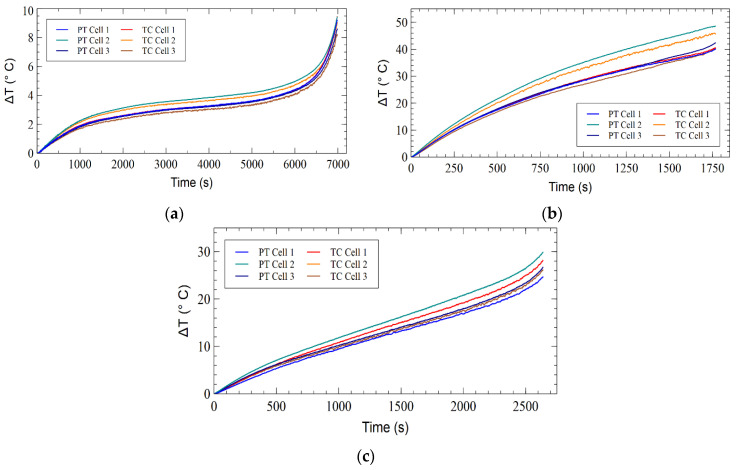
TC and PT temperature differential induced by (**a**) 0.5 C discharge, (**b**) 2 C discharge, and (**c**) 40 CW discharge.

**Figure 4:**
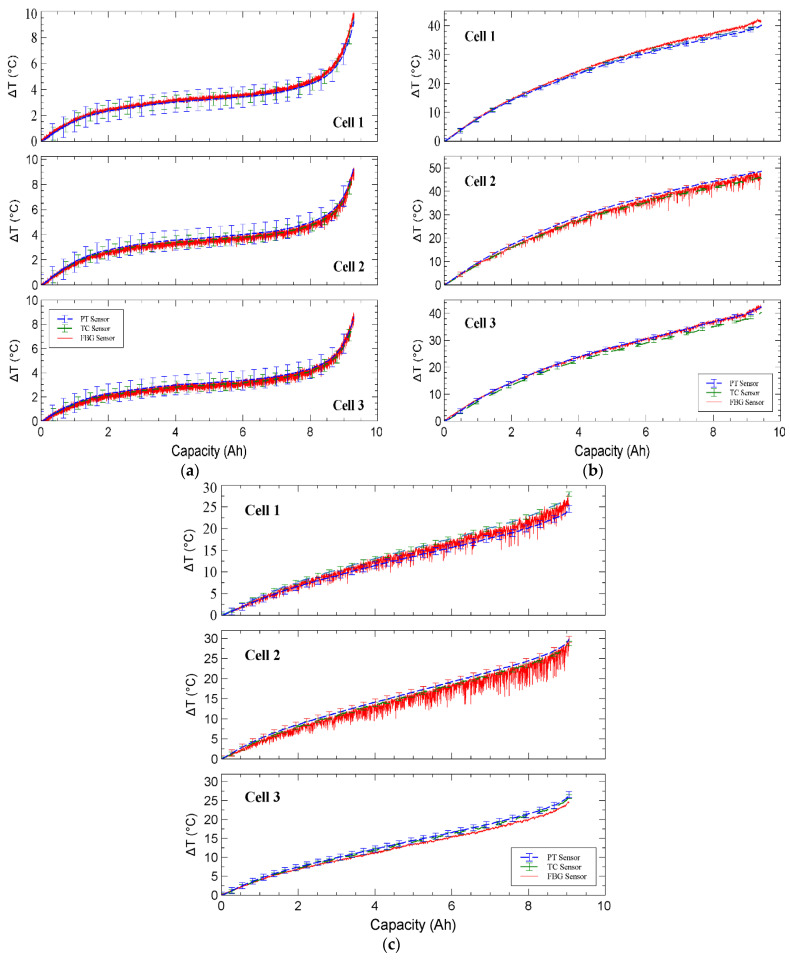
Comparison of sensor response for (**a**) 0.5 C, (**b**) 2 C discharge, and (**c**) 40 CW discharge. Plotted against capacity, each cell is shown individually, where FBG1, FBG 2, and FBG 3 are related to cell 1, cell 2, and cell 3, respectively.

Three discharge conditions induced an increase in surface temperature for each of the three LIB cells, which was measured by the three surface-mounted sensors. Figure 3 depicts the conventional TC and PT sensor response to the change in temperature of the cell surface. It was established that the sensor types exhibited the same trend for each of the discharge situations; furthermore, it demonstrated that cell 2 of the LIB pack had the highest temperature during the 2 C and 40 CW discharge conditions. Figure 4 depicts the total response of the three sensor types on the LIB cells, demonstrating that there was a high agreement between the FBGs and the TC and PT sensors for each cell. The 0.5 C discharge induced the lowest overall temperature change in the three discharge experiments. Figure 4a demonstrates that the FBGs showed some fluctuation over the discharge period; however, the FBGs on all three cells followed the temperature change over the discharge duration and fell within the PT and TC error bars. Figure 4b demonstrates that during the 2 C discharge, cell 2 had greater variance than the responses of FBG 1 and FBG 5 on cell 1 and cell 3, respectively. Figure 4c shows that FBG 3 varied below the TC and PT sensors during the 40 CW discharge, and in comparison, FBG 1 fluctuated between the PT and TC sensors, whereas FBG 5 fluctuated lower but closely follows the PT and TC sensors on cell 3.

Table 1 displays the maximum recorded temperature of each sensor for the three discharge conditions, allowing for a direct comparison of sensor types. Except for FBG 3 on cell 2, the sensors exhibited reasonable values for the 0.5 C discharge, i.e., the measurements were within a modest margin of each other. The data for 2 C revealed a more fluctuating scenario, with FBG 3 clearly showing the greatest temperature compared to FBG 1 and FBG 5, as well as a wider variance between each sensor, a scenario also visible for the 40 CW discharge albeit less pronounced. The measurements also showed that FBG 3 on cell 2 measured the greatest temperature, and, additionally, there is a greater fluctuation between the sensor types.

To thoroughly analyse the variance across sensor types during LIB discharge, the differences between the FBG and PT, FBG and TC, and, most critically, PT and TC were determined and are provided in Table 2. The variance between the PT and TC sensors must be evaluated as both are calibrated sensors, and any variation between them can be utilised to cross-reference the variation between the FBGs and conventional sensors, resulting in a robust representation of the FBGs’ performances. It can be seen throughout this dataset that the PT and TC variance is, in some cases, greater than that of the FBGs when compared to the conventional sensors; for example, the variation between the PT and TC sensors was greater than the FBGs on cell two and three during the 2C discharge and on cell 1 during the 40 CW discharge. In total, there were eight occasions where the FBG variances were lower than the variance of the PT and TC sensors. When comparing the variance between cells, the variation for the sensors on cell two was greater than both other cells on five of the six occasions and greater than one of the other cells on the other occasion. Furthermore, when comparing the PT sensor to the TC sensor, the variation in cell 2 was significantly greater during the 2 C discharge. It is also important to note that the biggest difference between the PT versus TC sensors was 3.26 °C, while the FBG was lower at 2.96 °C. When considering the response of the FBGs on each cell over the three discharge conditions, it was found that the average variation was 0.97 °C, 1.33 °C, and 1.27 °C for cells 1, 2, and 3, respectively. Overall, when placed in the guide tube mount technique, the FBGs were capable of delivering reliable temperature data comparable to traditional PT and TC sensors across the three-cell LIB.

### 3.2. Regression Analysis

Due to the high sensitivity of FBGs to strain and temperature, it was crucial to identify or quantify any forces of strain that the FBGs may have been subjected to while monitoring temperature. Strain forces influence the wavelength of the returning peak, which impacts the temperature measurement. Hence, a regression analysis was conducted to ascertain if straining force influenced the FBGs. As there was support for the FBGs at the extremities of the FOS strand, it was anticipated that their effects from straining forces would be less apparent or less than the FBG in the centre of the strand, which lacked mechanical support to avoid unintended bending. Regression analysis allowed for the correlation of the FBGs and conventional sensors, as well as the calculation of the standard error, allowing for comparison of FBG performance.

**Figure 5:**
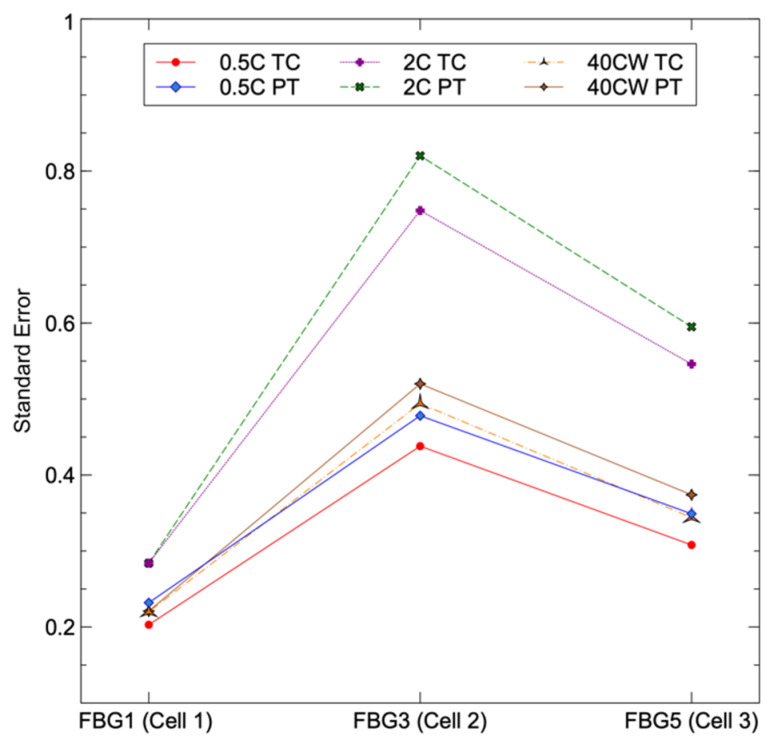
Linear regression analysis on standard error, with each traditional sensor compared to its respective FBG.

Table 3 shows the R^2^ and standard error of the linear regression analysis for each of the three cells where the FBGs are compared to conventional sensors. Figure 5 also includes a graphical representation of the standard error for each sensor. Since there are no directly correlated conventional sensors to compare with, the intermediate FBGs sensors were not taken into consideration for this regression analysis. The results show that under each discharge condition, the FBG, PT, and TC sensors exhibited a strong correlation, with R^2^ values exceeding 0.94. Cell 2 had the largest inaccuracy among the three FBGs examined in all discharge tests. FBG 1 had the lowest standard error for all three discharge situations, FBG 3 had the greatest standard error for all three discharge conditions, and FBG5 had a greater standard error than FBG1 but a lower standard error than FBG3. The findings confirmed the predicted theoretical hypothesis that mechanical support minimises uncertainty in peak wavelength measurements. Since FBG 1 had more mechanical support than FBG 5, the uncertainty was reduced. On the other hand, FBG 3 had the least support and the highest error of all of the FBGs.

### 3.3. Intermediate FBG Sensors

**Figure 6:**
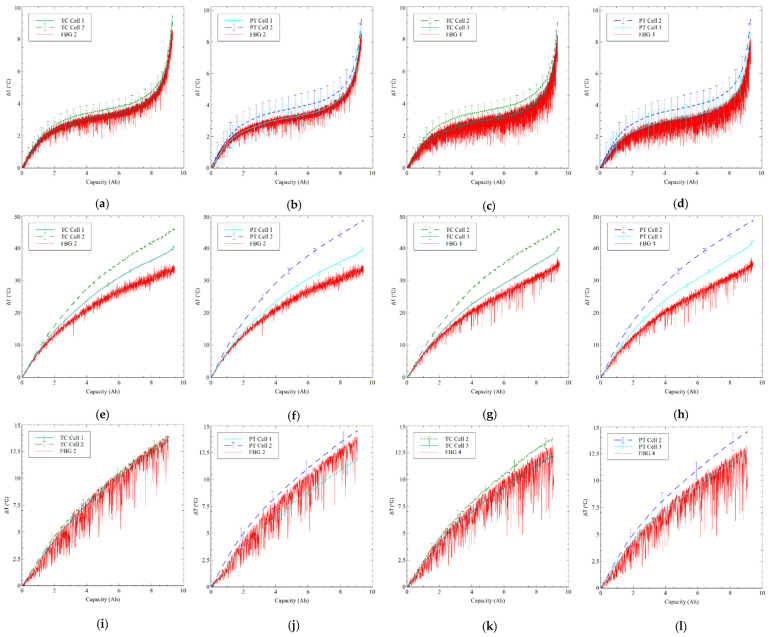
Comparison of FBG 2 and FBG 4 and relevant traditional sensors during discharge, where FBG 2 was situated between cell 1 and cell 2, and FBG4 was situated between cell 2 and cell 3. (**a**) 0.5 C FBG 2 with TC sensors on cell 1 and cell 2, (**b**) 0.5 C FBG 2 with PT sensors on cell 1 and cell 2, (**c**) 0.5 C FBG 4 with TC sensors on cell 2 and cell 3, (**d**) 0.5 C FBG 4 with PT sensors on cell 2 and cell 3, (**e**) 2 C FBG 2 with TC sensors on cell 1 and cell 2, (**f**) 2 C FBG 2 with PT sensors on cell 1 and cell 2, (**g**) 2 C FBG 4 with TC sensors on cell 2 and cell 3, (**h**) 2 C FBG 4 with PT sensors on cell 2 and cell 3, (**i**) 40 CW FBG 2 with TC sensors on cell 1 and cell 2, (**j**) 40 CW FBG 2 with PT sensors on cell 1 and cell 2, (**k**) 40 CW FBG 4 with TC sensors on cell 2 and cell 3, and (**l**) 40 CW FBG 4 with PT sensors on cell 2 and cell 3.

One of the most significant advantages of a FOS is the ability to combine several sensors into a single compact device. Since FBGs are extremely small, they can be positioned between cells, allowing for the detection of radiating heat at virtually no extra cost, which is not achievable with traditional PT and TC sensors. Moreover, a FOS can offer more information than a normal sensor. As demonstrated, five sensors can be connected with a single connection point, decreasing the complexity of the sensing system. Figure 6 compares the reaction of the PT and TC sensors on cells 1 and 2 to the temperature of FBG 2 placed between cells 1 and 2, as well as the temperature response of FBG 4, which is positioned between cells 2 and 3, to the corresponding PT and TC sensors on cells 2 and 3. When compared to the reaction of the 1 C discharge, the 2 C discharge results in a more significant rise in FBG 2 and FBG 4. Between cells 1 and 2, the void reaches 32.84 °C and 34.64 °C between cells 2 and 3. The 40 CW discharge responds to 1 C and 2 C discharge conditions, maintaining the pattern seen with the FBGs mounted on the LIB cells. The FBG response also exhibits higher variations but follows the trend of increasing temperature in all discharge conditions, supporting the use of these FBGs as a method to measure radiating heat from the LIB cells. The ability of the cooling approach to compensate for variations in the ambient temperature within the battery compartment could be advantageous for BTMS systems. Given that battery packs typically only provide limited information recorded at the module level, employing FBGs to record ambient temperatures in the battery compartment may be used to identify heat radiation from cells that have failed or are significantly degraded.

## 4. Conclusions

This study describes the deployment of a FOS strand with five FBG sensors for individual cell-level temperature monitoring of a 3-cell LIB pack. The usage of the PTFE guide tube with ABS 3D printed plinths produces high precision compared to the conventional PT and TC sensors. The regression data, especially the standard error, demonstrated that the FBG 3 response, located in the centre of the mounted FOS strand, exhibited a more variable response. The higher inaccuracy found throughout the three discharge tests might suggest a slack in the FOS that is just mildly interfering with the temperature measurements. A possible future solution would be to apply a method of supporting the FBG around the cell without interfering with the FBG located at the cell surface. Additionally, a mechanical method that applies slight tension to the FOS strand across the cells would minimise slack in the FOS, but this must be carefully designed to allow for the expansion and contraction of the entire FOS strand to decouple the strain and temperature. It has been demonstrated that, in addition to measuring the temperature at the cell level, temperature data can be captured between cells, as demonstrated by the temperature response of FBG 2 and FBG 5 for each discharge, where a lower temperature was recorded in comparison to the FBGs located at the cell surface. Due to the small dimensions and minimally invasive nature of FBG sensors, FBGs may be placed into the small gaps between cells, thus the greater fluctuation detected by these FBG sensors in this study might be utilised to monitor and examine the heat radiated from cells within a battery pack in the future.

## Figures and Tables

**Table 1:** The maximum temperature recorded by each sensor during three discharge conditions.

Sensor (Discharge)	Cell 1	Cell 2	Cell 3
FBG (0.5 C)	34.08	33.55	32.88
PT (0.5 C)	34.09	34.42	33.05
TC (0.5 C)	34.55	34.03	33.58
FBG (2 C)	66.33	72.53	67.31
PT (2 C)	64.98	73.54	67.38
TC (2 C)	65.55	70.59	65.62
FBG (40 CW)	50.27	52.53	48.87
PT (40 CW)	49.97	54.90	51.06
TC (40 CW)	53.23	53.84	51.68

All data are in degrees Celsius (°C).

**Table 2:** Variation in maximum recorded temperature.

Sensor (Discharge)	Cell 1	Cell 2	Cell 3
FBG vs. PT (0.5 C)	−0.01	−0.87	−0.17
FBG vs. TC (0.5 C)	−0.47	−0.48	−0.70
PT vs. TC (0.5 C)	−0.46	0.39	−0.53
FBG vs. PT (2 C)	1.35	−1.01	−0.07
FBG vs. TC (2 C)	0.78	1.94	1.69
PT vs. TC (2 C)	−0.57	2.95	1.76
FBG vs. PT (40 CW)	0.3	−2.37	−2.19
FBG vs. TC (40 CW)	−2.96	−1.31	−2.81
PT vs. TC (40 CW)	−3.26	1.06	−0.62

All data are in degrees Celsius (°C).

**Table 3:** Linear Regression Data.

Discharge	Sensor	Cell 1 (FBG 1)		Cell 2 (FBG 3)		Cell 3 (FBG 5)	
		R^2^	St Error	R^2^	St Error	R^2^	St Error
0.5 C	PT	0.99	0.23	0.98	0.48	0.99	0.35
	TC	0.99	0.20	0.98	0.44	0.99	0.31
2 C	PT	0.99	0.28	0.99	0.82	0.99	0.59
	TC	0.99	0.28	0.99	0.75	0.94	0.55
40 CW	PT	0.98	0.22	0.98	0.52	0.99	0.37
	TC	0.98	0.22	0.98	0.49	0.99	0.34

## Data Availability

Not applicable.
